# The impact of an ambulance vehicle preparation service on the presence of bacteria: a service evaluation

**DOI:** 10.29045/14784726.2019.03.3.4.27

**Published:** 2019-03-01

**Authors:** Mo Mackenzie, Richard Pilbery

**Affiliations:** Yorkshire Ambulance Service NHS Trust; Yorkshire Ambulance Service NHS Trust

**Keywords:** ambulance vehicle preparation, ATP swabs, infection prevention and control

## Abstract

**Introduction:**

Around 300,000 patients a year in England acquire a healthcare-associated infection (HAI) while being cared for by the NHS. The contribution from NHS Ambulance Services is not known, but previous studies have identified the presence of pathogenic bacteria such as Methicillin-resistant *Staphylococcus aureus* (MRSA) and *Enterococcus*, including resistant strains in some cases, inside ambulances. To improve ambulance cleanliness, Yorkshire Ambulance Service NHS Trust (YAS) piloted an Ambulance Vehicle Preparation Service (AVPS) at two ambulance stations, where staff were tasked with ensuring every ambulance at these stations was cleaned every 24 hours.

**Methods:**

Adenosine triphosphate (ATP) bioluminescence testing was conducted on 16 ambulances at the two pilot AVPS stations and on 18 ambulances at four ‘business as usual’ (BAU) ambulance stations using a Hygiena SystemSURE luminometer. Swabs were obtained from 10 pre-selected locations inside each ambulance.

**Results:**

Between November 2016 and August 2018, a total of 690 swabs were obtained and recorded from 34 ambulances. Overall, median relative light unit (RLU) values for both groups were < 100, with only the BAU group having an upper quartile value > 100. However, when stratified by swabbing area, three areas had a median RLU of > 100 in the BAU group: suction unit handle, steering wheel and airway seat shelf. In addition, the upper quartile RLU values for the grab rail above the stretcher and the passenger seat in the BAU group were also > 100. No swab areas had a median RLU > 100 in the AVPS group.

**Conclusion:**

A dedicated AVPS results in better cleaning of ambulance vehicles than the existing cleaning system utilising operational crews. The areas most likely to be contaminated are the suction unit handle, steering wheel, airway seat shelf and grab rails. The position of equipment and the materials that equipment are constructed from should have infection prevention and control (IPC) as a consideration.

## Introduction

It is estimated that around 300,000 patients a year in England acquire a healthcare-associated infection (HAI) while being cared for by the NHS (National Institute for Health and Care Excellence, 2014). The contribution from NHS Ambulance Services is not known, but previous studies from Wales, Europe and the United States have identified the presence of pathogenic bacteria such as Methicillin-resistant *Staphylococcus aureus* (MRSA) and *Enterococcus*, including resistant strains in some cases inside ambulances (Alves & Bissell, 2008; Eibicht & Vogel, 2011; Makiela, 2016; Nigam & Cutter, 2003; O’Hara et al., 2017; Vikke & Giebner, 2016).

### Ambulance vehicle preparation service

In response to growing concerns about infection prevention and control (IPC) in the Yorkshire Ambulance Service NHS Trust (YAS), and the issue of insufficient time at the commencement of shifts to conduct vehicle cleaning and checking, the Trust introduced an Ambulance Vehicle Preparation Service (AVPS) at two ambulance stations in February 2016. Staff were employed and trained to conduct a vehicle preparation process on all ambulances based at their respective ambulance stations, every 24 hours. This vehicle preparation process includes:

- refuelling and external cleaning;
- internal cleaning;
- consumables (e.g. oxygen masks, cannulas) stock and expiry date checking; and
- medical equipment and devices checking, testing and cleaning.

An AVPS team can complete all vehicle preparation tasks in approximately 50 minutes per double crewed ambulance (DCA). This process ensures that every item of medical equipment, and all medical devices, are clean and fully functional, and the vehicle is appropriately stocked with consumables to last for a 24-hour period of patient care.

In contrast to the AVPS is the Trust’s ‘business as usual’ (BAU) ambulance cleaning. The BAU cleaning forms part of the vehicle checking process that is expected to occur in the first 20 minutes of a shift. This is undertaken by the ambulance crew working on the vehicle and is subject to potential interruption if an emergency call is allocated to the crew in this time.

In order to determine the effectiveness of the AVPS at vehicle cleaning, an evaluation was conducted to compare the cleanliness of vehicles prepared by the AVPS team to BAU ambulance cleanliness.

## Methods

Two pilot AVPS stations and four BAU ambulance stations were selected for the evaluation. BAU stations were based on their similarity with the AVPS pilot locations, i.e. busy urban ambulance stations. Adenosine triphosphate (ATP) bioluminescence testing was conducted on 16 ambulances at the two pilot AVPS stations and on 18 ambulances at four BAU ambulance stations using a Hygiena SystemSURE luminometer. ATP is the principal energy carrier for all living organisms, including bacteria, and ATP testing is widely used in the food industry and healthcare settings to measure the effectiveness of cleaning (Shama & Malik, 2013). Bioluminescence tests work by creating a chemical reaction between the ATP and an enzyme, luciferase, which generates light that can be measured by a test device. The level of bioluminescence is proportional to the amount of ATP present and is expressed in relative light units (RLU). It has several advantages over more traditional aseptic swab testing and culturing, including being inexpensive and quick to perform.

While there is no clear guidance about what constitutes an acceptable RLU value for ambulances, a value of 100 RLU was chosen, based on a previous study that correlated RLU with potentially harmful microbial growth levels, and manufacturer’s guidance (Mulvey et al., 2011).

An agreed set of specific areas to be swabbed was chosen by the head of safety and the IPC lead for YAS based on a National Ambulance Service Infection Prevention and Control Group (NASIPCG) project. The locations were a mixture of both low and high traffic areas. The ambulance trolley was excluded, since it was originally anticipated that swabbing would take place at hospitals while crews were handing over their patients, and there was concern about avoiding an operational delay. The sites were not discussed with any persons involved in ambulance vehicle cleaning, either within the AVPS or Ancillary Services. This included 10 swab sites on each vehicle:

1. Laerdal Suction Unit (LSU) rubberised handle;
2. medicine cupboard and safe door;
3. grab rail above the ambulance stretcher;
4. steering wheel;
5. passenger seat;
6. wall behind the sharps bin;
7. inside the response bag storage cupboards;
8. inside the splint storage cupboards;
9. under the carry chair seat; and
10. shelf by the airway seat.

To ensure a fair comparison, swabbing of ambulances occurred at the following time points:

- At AVPS stations, swabs were obtained after the ambulance had been cleaned by the AVPS crews, but prior to being taken out on shift by an operational ambulance crew.
- For BAU stations, swabs were obtained after the ambulance crew had completed their vehicle checks and undertaken any cleaning they felt appropriate prior to being called out to an incident.

Ambulances were not swabbed if they had just returned from deep cleaning (a process conducted every 35 days, involving stripping the vehicle of all its medical equipment, devices and consumables and ensuring that everything is individually cleaned: a four-hour process) and had not yet been out on operational duties, or if they had just been returned from fleet, or been ‘off-the-road’ and had not yet been checked by an operational crew at BAU stations.

The swabbing was conducted according to the manufacturer’s instructions and was identical for all pre-specified sites.

The AVPS crews had the same guidance for vehicle cleaning as the BAU crews, with both groups expected to follow the procedure specified in the Trust’s document, ‘Decontamination of Medical Devices and Vehicle Procedure’.

## Results

Between 11 November 2016 and 7 August 2018, ATP testing was conducted on 16 ambulances at the two pilot AVPS stations and on 18 ambulances at 4 BAU ambulance stations. A total of 690 swabs were obtained and recorded from 34 ambulances (Table 1). Due to the integration of the AVPS within the hub and spoke model introduced at the time of the evaluation, more AVPS vehicles were available to be swabbed than BAU vehicles.

**Table 1.** Summary information for evaluation.

Variable	AVPS	BAU	All
Swab events	470	220	690
Swabbed ambulances	16	18	34
Median days since last deep clean (IQR)	15 (4–24)	13.5 (6–22)	15 (4.5–22)
Median RLU (IQR)	10 (3–35)	31 (7–130)	13 (4–50)

Overall, median RLU values for both groups were < 100, with only the BAU group having an upper quartile value > 100. Median RLU values were lower in the AVPS group, both overall and in every single swab location (Table 1). Note that since AVPS vehicles were more frequently available, it was possible to swab them more often than the BAU vehicles.

**Figure 1:**
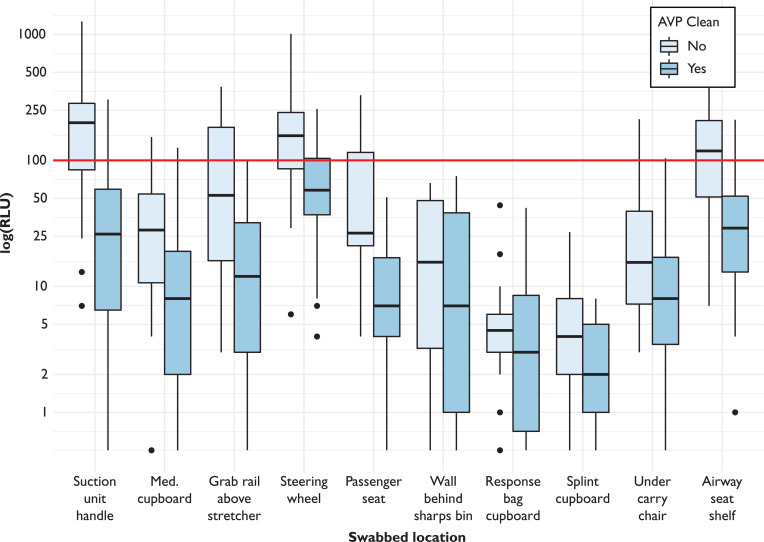
Figure 1. Boxplot of swab results, stratified by swab location and whether cleaning conducted by AVPS.

However, when stratified by swabbing area, some areas for concern became apparent (Table 2 and Figure 1). Three areas had a median RLU of > 100 in the BAU group: suction unit handle, steering wheel and airway seat shelf. In addition, the upper quartile RLU values for the grab rail above the stretcher and the passenger seat in the BAU group were also > 100. No swab areas had a median RLU > 100 in the AVPS group, although the steering wheel had the highest median value (58), and had an upper quartile value of 104.

**Table 2.** Swab RLU results stratified by location and AVPS cleaning.

	APVS		BAU	
*Swab location*	*Median RLU (IQR)*	*Maximum RLU*	*Median RLU (IRQ)*	*Maximum RLU*
Suction unit handle	26 (6.5–59)	304	199 (80–293)	1265
Medicine cupboard	8 (2–19)	126	28 (10–62)	153
Grab rail above stretcher	12 (3–32)	100	53 (16–186)	384
Steering wheel	58 (37–104)	256	157 (75–240)	1010
Passenger seat	7 (4–17)	51	26.5 (21–121)	329
Wall behind sharps bin	7 (1–39)	75	16.5 (3–49)	66
Response bag cupboard	3 (0.5–8.5)	42	4.5 (3–6)	44
Splint cupboard	2 (1–5)	8	4 (2–8)	27
Under carry chair	8 (3.5–17)	104	15.5 (7–40)	212
Airway seat shelf	29 (13–52)	210	119.5 (51–207)	737

## Discussion

The results of this evaluation clearly show that the median RLU values in all areas were lower in the AVPS group compared to the BAU group. In addition, the interquartile ranges (IQR) for all swabbing sites (with the exception of the steering wheel) in the AVPS group were under the safe threshold of 100 RLUs. This contrasts with the BAU group, where the suction unit handle, steering wheel, passenger seat and airway seat shelf all recorded an IQR that exceeded the 100 RLU threshold. The BAU group also recorded the highest RLU values, with two sites (suction unit handle and steering wheel) recording a maximum RLU of over 1000.

While ATP testing does not identify the pathogen, and whether it is dangerous to health, other studies have identified antimicrobial resistant strains of bacteria in ambulances. Wepler et al. (2015) conducted unannounced visits at 56/78 of German emergency ambulance stations, collecting samples from 150/225 (67%) of ambulances. Of the samples collected, 5% contained evidence of contamination by MRSA. Non-resistant strains of *S. aureus* and *Enterococcus faecalis* have been detected on Danish ambulances by Vikke and Giebner (2016), who randomly swabbed cleaned blood pressure (BP) cuffs from Danish ambulances. The contamination was thought to be due to improper cleaning and/or cross-contamination after cleaning. While the risk to patients tends to be at the forefront when trying to ensure good IPC practices, it should not be forgotten that staff are just as easily exposed to the same pathogens, potentially spreading them to other patients and colleagues, causing illness and time off work.

The location of contamination perhaps unsurprisingly centred on areas and equipment that were frequently used and therefore exposed to contamination. These included carrying handles, ECG cables, BP cuffs, ambulance stretchers and carry chairs, as well as seatbelts and seatbelt release buckles (Eibicht & Vogel, 2011; Makiela, 2016; Vikke & Giebner, 2016; Wepler et al., 2015). Contamination into the ambulance cab was also identified by Alves and Bissell (2008). While they did not swab the steering wheel, they did find pathogens on the driver’s door interior grip.

The rubberised handle of the LSU raises the question about whether the texture and material that medical devices are constructed from might affect whether contamination is more likely to occur. For example, textured oxygen knobs have been found to have high levels of contamination (Alrazeeni & Al Sufi, 2014). The BP cuffs used in one Danish study supposedly had antimicrobial protection built into the fabric, but *S. aureus* and *Enterococcus* were found post-cleaning (Vikke & Giebner, 2016).

It is important to remember that both the AVPS and BAU crews were following the same guidance for cleaning ambulances and equipment, which makes it all the more significant that the results were so different. There are a couple of explanations for this. The first, most credible, is that AVPS staff received comprehensive training on IPC and vehicle cleaning, which was monitored by way of frequent auditing of the cleaning process. The second could be due to one of the main motivations for conducting this evaluation in the first place: the concern that crews did not have enough time to undertake cleaning during the initial vehicle check and the commencement of their shift.

While not explored in this evaluation, it is possible that there are other benefits to having an AVPS service. For example, AVPS staff ensured that the correct stock of consumables was on the ambulance. Anecdotally, over-stocking of consumables can be a problem. Restocking and vehicle preparation conducted by AVPS staff may also result in a time-saving for operational crews, who may not have to spend so long checking their vehicles and engaging in vehicle cleaning.

### Limitations

Utilising ATP testing instead of aseptic swabbing meant that it was not possible to identify the actual pathogens present, or how harmful they were to patients and staff. This was a pragmatic decision, since the logistics and cost of using a laboratory-based testing service made it impractical to implement in a busy ambulance service. In addition, ATP testing accuracy can be adversely affected by the presence of cleaning/disinfecting chemistry residues, resulting in artificially low readings (Omidbakhsh, Ahmadpour, & Kenny, 2014). In retrospect, it might have been beneficial to have swabbed other areas, for example the BP cuff, to enable more of a comparison with existing literature.

## Conclusion

A dedicated AVPS results in better cleaning of ambulance vehicles than the existing cleaning system utilising operational crews. The areas most likely to be contaminated are the suction unit handle, steering wheel, airway seat shelf and grab rails. The position of equipment and the materials that equipment are constructed from should have IPC as a consideration.

## Conflict of interest

None declared.

## Ethics

Not required.

## Funding

None.
